# Segmentation and Multi-Scale Convolutional Neural Network-Based Classification of Airborne Laser Scanner Data

**DOI:** 10.3390/s18103347

**Published:** 2018-10-07

**Authors:** Zhishuang Yang, Bo Tan, Huikun Pei, Wanshou Jiang

**Affiliations:** 1 State Key Laboratory of Information Engineering in Surveying, Mapping and Remote Sensing, Wuhan University, Wuhan 430010, China; tcyzs@whu.edu.cn; 2 Shenzhen Power Supply Co., Ltd., No. 2018 Cuizhu Road., Shenzhen 430079, China; sztanbo@126.com (B.T.); peihuikun@sz.csg.cn (H.P.)

**Keywords:** region growing segmentation, multi-scale convolutional neural network, ALS point clouds, semantic 3D labeling, feature image

## Abstract

The classification of point clouds is a basic task in airborne laser scanning (ALS) point cloud processing. It is quite a challenge when facing complex observed scenes and irregular point distributions. In order to reduce the computational burden of the point-based classification method and improve the classification accuracy, we present a segmentation and multi-scale convolutional neural network-based classification method. Firstly, a three-step region-growing segmentation method was proposed to reduce both under-segmentation and over-segmentation. Then, a feature image generation method was used to transform the 3D neighborhood features of a point into a 2D image. Finally, feature images were treated as the input of a multi-scale convolutional neural network for training and testing tasks. In order to obtain performance comparisons with existing approaches, we evaluated our framework using the International Society for Photogrammetry and Remote Sensing Working Groups II/4 (ISPRS WG II/4) 3D labeling benchmark tests. The experiment result, which achieved 84.9% overall accuracy and 69.2% of average F1 scores, has a satisfactory performance over all participating approaches analyzed.

## 1. Introduction

In processing digital terrain models (DTM) and 3D city and landscape models, point clouds have become a more and more popular type of data. For photogrammetry, common point clouds can be produced by airborne laser scanning (ALS) [1,2] and by dense matching of aerial photographs [3]. No matter which method is chosen, the classification of point clouds cannot be ignored. It is the first step in extracting productive geo-information. In some productions, such as DTM generating, points only need to be classified into two classes. In other processes, such as city reconstruction, points require classification into multiple categories. Some existing classification tasks are implemented on a point-based method, while other works proposed to use a segment-based method [4]. Point-based methods use the information of each point with reference to its neighbor, such as eigenvalue-base features, point density values, and the direction of normal vector, or information based on the point itself, such as intensity value and echo-based features, to obtain accurate classification results. On the contrary, segment-based methods divide the point cloud into segments first and put the class label into each segment within which all points belong to the same category.

Admittedly, segment-based classification methods outperform point-based methods in some respects. Above all, a segment-based method is always a timesaving method. The number of segments is much smaller than the number of points. Although dividing point clouds into segments may initially take some time, segment-based methods put a label into each segment which contains hundreds of points. The time of generating features and labeling will then be reduced. Secondly, segments may contain more extending features than a single point within its local neighborhood. Features such as segment size, segment point density, and average echo number in a segment can then be used. The categories’ separability may be improved by these features.

The advantages of segment-based classification cannot be realized without good segmentation. Under- and over-segmentation errors will negatively affect the classification accuracy [5]. Undeniably, under-segmentation will cause classification errors as all the points in a segment belong to the same category. Meanwhile, over-segmentation will add computational effort and reduce the reliance of the segment-based features.

In this paper, a segment-based method is used to reduce the computational burden of our previous work [6], which is a point-based convolutional neural network labeling method. The scientific contributions of this study are as follows:

- We propose a three-step region-growing segmentation method for segment-based classification. We divide the segmentation into three steps in order to provide a good starting point for the following procedure.
- We also develop our convolutional neural network. A multi-scale convolutional neural network is trained to automatically learn deep features of each point from the generated feature images across multiple scales.

The paper is structured as follows: the related work to this subject is discussed in Section 2. We present our methodology in Section 3. Section 4 presents the experimental results. The results from using our method is compared with those found using state-of-the-art segment-based and point-based methods. A discussion of our experiments is in Section 5. We provide concluding remarks and suggestions for future work in Section 6.

## 2. Related Work

In order to understand the relationship between data and labels, some modern discriminative methods are provided. Adaboost [7], support vector machine (SVMs) [8], random forests (RFs) [9], conditional random fields (CRFs) [10], and deep convolutional networks (DCNNs) [11] are popular ones. These methods are also used for the ALS data. The classification methods of the ALS data can be divided into two categories: the point-based classification and the segment-based classification [5].

For point-based classification, an Adaboost [12] algorithm, which automatically combines rough guesses to a more accurate hypothesis, was used to label the 3D ALS data into four classes. Five features were used in the classification. A SVM classifier was used in Mallet’s work [13]. It is a point-based method for LiDAR data. SVM is a non-parametric method. It performs well especially in non-linearly separable data. The potential of LiDAR data is developed by using the SVM method. Chehata [14] used the RF method to classify the LiDAR data. Random forests can make full use of the multi-echo and the full-waveform LiDAR features and provide an accurate classification result in an efficient way, even if the datasets are large. For a thorough discussion of supervised classifiers, Weinmann [15] applied 10 different methods to a same procedure to evaluate their performance. Both methods treat each point independently. In more complex places, such as urban areas, this drawback may lead to inhomogeneous results, as mentioned in Niemeyer’s work [16]. In urban areas, many different objects appear in even a small scene. Roofs and other challenging objects, like cars, fences, and hedges, may have many details, causing overlapping distributions of features in each class. Errors such as shadows caused by other objects, missing data, and random errors make the problem bigger.

In order to overcome these problems, the contextual information that contains the relationship between 3D points within a neighborhood is introduced into the classification of ALS data. The relationships between the object classes can be trained to make the results better. For example, a facade is more likely to appear next to a roof, and a fence is more likely to appear on top of grass. Probabilistic graphical models, such as the conditional random field model, are used for that reason. Niemeyer [17] presented a point-based CRF classifier for urban ALS data. He used a graphical model to represent the point cloud. The edges of the graphical model link each point to its 2D neighbors. The relationships between object categories and the datasets are learned in a training step by making use of a complex model. By comparing the classification result to the methods without contextual information, the CRF method achieves a smoother and more accurate result, even for the classes that come in a low quantity like garages and pavilions.

There are also some problems in the pairwise CRF method. The interactions only occur at a very local level. Thus, some isolated clusters of points may be classified into wrong classes. Many researchers have improved the CRF method to handle these missing long-range interactions. Luo and Sohn [18] presented a multi-rand and asymmetric conditional random field (maCRF). In maCRF, prior information of scene-layout compatibility is used to handle the long-range dependency problem. The maCRF combines two CRF models. One is the short-range CRF for the local neighborhood, and the other is a long-range CRF for the long-range interactions. The final results are refined by independently using the output of the two models. Another solution is proposed by Xiong [19]. A multi-stage inference procedure is used to handle the difficulties in modeling the contextual relationships between the 3D points. A segment result is achieved by using a point-based classification first. Then, the contextual information is presented using the segment result for the final point-based classification. This P^N^ Potts model is proposed by Kohli [20]. The mutually propagating and iterating contextual information improved the classification results. Local spatial interactions can be restricted by the P^N^ Potts model. In a large scale, some potential misclassification may be revised.

The convolutional neural networks also take the contextual information into consideration for point-based classification tasks. Boulch [21] picked several snapshots of the point cloud. For each snapshot, an RGB and geometric composite image was generated. The 3D data was then transformed into 2D images. The fully convolutional neural network was trained by these images and used for pixel-wise labeling. Caltagirone [22] applied a simple and fast fully convolutional neural network (FCN) to assist with road detection. Top-view images encoding several basic statistics, such as mean elevation and density, were generated. The FCN is specifically designed for the task of pixel-wise semantic segmentation by combining a large receptive field with high-resolution feature maps. Yousefhussien [23] presented a 1D FCN to generate point-based labeling while implicitly learning contextual features in an end-to-end fashion. Yang [6] presented point-based feature image generation for the CNN. For each point in the ALS data, a neighboring point with a window was extracted. Using their point-based features, the feature images containing the contextual information were then generated. Relationships between the point and the feature image were learned by the CNN model.

Another way to improve the labeling results and reduce time cost is segment-based classification. More stable features can be achieved since segments may contain some extending features compared to a single point within its local neighborhood. Furthermore, the number of segments is much smaller than the number of points, so time will be saved even though the segmentation process is added. Golovinskiy [24] presented a system for detecting objects such as traffic lights or cars using the combined terrestrial and ALS data. First, the potential object locations were determined based on a hierarchical clustering method. Then, a graph-cut-based segmentation was applied to classify the points close to these locations into the foreground and background. The segmentation method required the parameters of the segments, such as the maximum radius, to be set in advance. The points in the foreground segments were treated as objects while the points in the background segments were discarded. The feature vectors were calculated based on context and shape information and applied to a classifier. Shapovalov [25] used the k-means method to perform the segmentation. Each point was treated as a leaf of a tree and a heuristic method was used to reduce the computational burden. Since the k-means method only defines the total number of segments, it leads to strong over-segmentation. A graph over medoids of segments was built, and the edge values were determined by analyzing the k-nearest neighbors of the medoids. A naïve Bayes classifier was used to define the pairwise potentials. Features such as deviations of the surface normal of the segments and the geometrical arrangement of the medoids were considered. The experiment result showed that the segment-based method can remove noise, increase efficiency, and make use of natural edge features. In Xu’s work [4], single points and two types of segments acquired by different methods were treated as entities for the classification method. Features such as z variance, distance ratio, segment size, and normal direction were calculated from these three entities. The classification was based on heuristic rules, and the contextual information was considered using the segment-based methods. Niemeyer [26] merged the spatial and semantic context in a two-layer CRF. The output of the first CRF was used to generate segments. The segments contained larger scale context and was introduced as an energy term for the next iteration of the next CRF layer. Guinard and Landrieu [27] proposed a non-parametric segmentation model for the classification of 3D LiDAR point clouds in urban areas. The high-level structure of the area was captured by integrating the segmentation into the CRF. The segment-based method aggregated the noisy predictions of a weakly-supervised classifier and produced a higher accuracy result. Vosselman [5] thought that different segmentation methods may be good at different object classes. Thus, a hierarchical structure containing two different segmentation methods was proposed to obtain a generic technique for the ALS data. The structure was capable of handling complex urban areas with a large number of categories. The combination of small and large segments produced by the hierarchical structure made the interaction between nearby and distant points possible. The contextual information was learned by using a CRF. The edge value of the graph was defined by the boundaries of the segment rather than the medoids [25]. The features extracted by analyzing the boundary of the segment were added to improve the classification accuracy.

This paper is based on our previous work [6]. We changed the point-based method to a segment-based method. A three-step region-growing method was proposed for the segmentation. Feature images in different scales were generated and these feature images were treated as the input of a multi-scale convolutional network, and the CNN model was trained for the final semantic labeling task.

## 3. Methodology

### 3.1. Three-Step Region-Growing Segmentation

**Figure 1:**
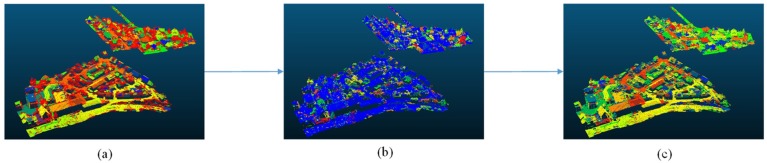
The three-step region growing method: (**a**) planar object extraction, (**b**) small pieces re-segmentation, and (**c**) merging.

A three-step region-growing method was used for the segmentation, as shown in Figure 1. Normal direction, echo intensity values, and the planarity were key parameters in these steps. The basic region-growing method that we use was developed by Rabbani [28]. In order to obtain a proper segmentation result, we chose to use a three-step region-growing method. Different point values were used in each step.

In the first step, we used the region-growing method to find planar objects. We first sorted all the points by their curvature. The region began its growth from the point that had the minimum curvature since growth from the flattest areas allows for the reduction of the total number of segments [28]. Then, local surface normal vectors and echo intensity values were used to cluster the points. The local plane ($\Pi_{P}$) was calculated using an M-estimator [29]. The normal direction for each point was calculated by fitting the plane to some neighboring points. Some objects such as low vegetation and impervious surfaces were hard to separate using only normal vectors. The chosen echo intensity values were high on building roofs, on gravel roads, and on cars, while low values were asphalt roads and tar streets [14]. This could solve this problem and enforce the segmentation. If the differences of the normal angles and the intensity values between the point and its neighbor were beneath the threshold, the point was added to the segment. The threshold was made to be small so that planar objects such as roofs and walls were in large segments. Other objects, such as cars and trees, were in small pieces. Then, segments beneath a certain size were discarded and the removed points were re-segmented in the second region-growing step.

In the second step, we used the region-growing method to get smaller objects, such as cars, shrubs and trees. We also set our growth to begin from the point with the minimum curvature to reduce the total number of segments. Then, the point planarity value was used to cluster the points. In the point clouds, the center of gravity was written as $\bar{X}=\frac{1}{n}\sum_{i=1}^{n}X_{i}$. The vector $M=\left(X_{1}-\bar{X},\cdots ,X_{n}-\bar{X}\right)$ was defined. Then, we calculated the variance-covariance matrix as:

(1) $$WC=\frac{1}{n}M^{T}M$$

From the matrix, the eigenvalues $\lambda_{1}$ > $\lambda_{2}$ > $\lambda_{3}$ were calculated. Additional features [30] were descried as follows:

(2) $$\mathrm{Planarity}:\;P_{\lambda }=\frac{\lambda_{2}-\lambda_{3}}{\lambda_{1}}$$

If the differences of the planarity values between the point and it neighbor were beneath the threshold, the point was added to the segment. Segments beneath a certain size were discarded too. Since most points on trees or cars were grouped to single segments, fewer points remained unsegmented.

Finally, the unsegmented points were merged into the most frequent segments in their neighbor region. We used a Kd-tree [31] structure for finding the nearest neighbor points. All the points then had a segment label.

### 3.2. Feature Image Generation

For features selection, we chose features stated in [6] which may be useful for the classification result. The following features were used as shown in Table 1.

To find the height above DTM, the DTM was generated using robust filtering [32], which is implemented in the commercial software package SCOP++. It helped to distinguish categories since it can reflect the global distribution for a point. This feature is, by far, the most important since it is the strongest and most discernable feature for all the categories and relationships based on the analysis by Niemeyer [17]. The description of echo intensity values and planarity were included in Section 3.1, and the sphericity can be calculated as follows:

(3) $$\mathrm{Sphericity}:\;S_{\lambda }=\frac{\lambda_{3}}{\lambda_{1}}$$

The variance of deviation angles can be calculated using the angle between the point normal vector and the vertical direction. This feature can help us separate planar surfaces such as roads from vegetation [6]. An eigenentropy-based scale selection method [15] was used to determine the neighborhood scale for computing these features. In total, all five features were used to generate the feature image. The echo intensity values were scaled to the range of 0–255, and the other four features were scaled to the range of 0–1.

Feature images were generated based on these features. For each point in the ALS data, a square window was set up. The point was located at the center of the window, and the window is parallel to the x–y plane. The window was divided into 128 × 128 cells. The center of each cell is calculated as:

(4) $$\{\begin{array}{c} X_{i,j}=X_{p}-(63.5-j)\times w\\ Y_{i,j}=Y_{p}-(63.5-i)\times w\\ Z_{i,j}=Z_{p} \end{array}$$

*i* and *j* denote the row and column number; $X_{p}$, $Y_{p}$, and $Z_{p}$ denote the coordinates of the point; and w is the width of the cell.

If the width of the cell is small, the feature image may contain a lot of white pixels (pixels do not contain any point). This may influence the classification result in next step. Thus, we chose to find the nearest point around each cell center and assign features to it, even if the point was not in the cell. Five features were transferred into three integers as shown in [6]:

(5) $$\begin{array}{c} RED=\left[255\times S_{\lambda }\times \sigma_{z}^{2}\right]\\ GREEN=\left[Intensity\times P_{\lambda }\right]\\ BLUE=\left[255\times H_{above}\right] \end{array}.$$

**Figure 2:**
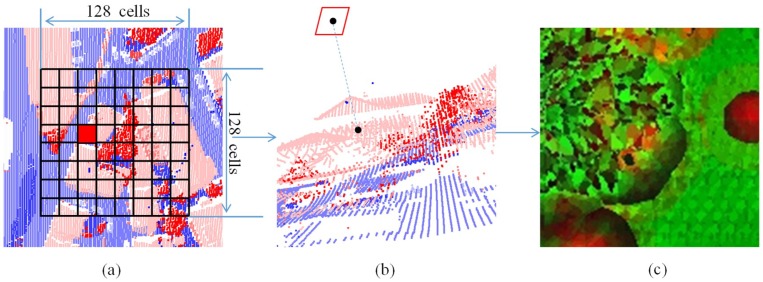
The steps of feature image generation: (**a**) Set up the square window and find the cell. (**b**) Search for the nearest point and assign the value to the cell. (**c**) Generate the feature image.

The steps of feature image generation are shown in Figure 2.

### 3.3. The Multi-Scale Convolutional Neural Network (MCNN)

**Figure 3:**
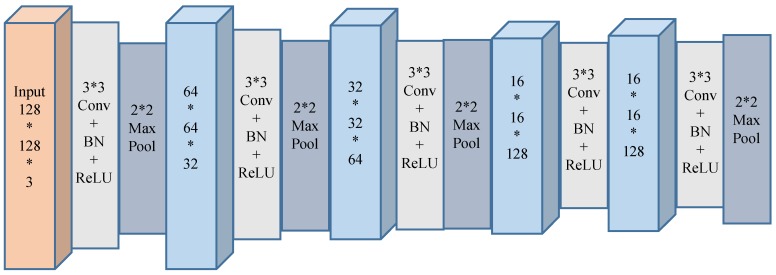
The architecture of the SCNN.

The MCNN was implemented with Caffe [33]. The MCNN consists of three single-scale convolutional neural networks (SCNNs). The architecture of the SCNN is shown in Figure 3. The SCNN is comprised of four kinds of layers. A detailed explanation of the layers is as follows.

In Figure 3, “Conv” denotes the convolutional layer, which is the most frequent layer. It performs the convolution operation with the weight of the network. In our model, the size of the convolutional kernels is 3 × 3. “Pool” denotes the pooling layer, which can reduce the number of parameters to be learned and improve the robustness of the translation. In our model, the max-pooling strategy is used. BN denotes the batch normalization layer and the ReLU denotes the rectified linear unit layer. BN can improve the learning rate and reduce overfitting [34]. ReLU can improve the learning rate and eliminate the need for unsupervised pretraining during the training of a deep supervised network [35].

Using a single scale image patch to represent a point is problematic when the point has a relative complex surrounding environment. In order to obtain enough semantic information and make a more precise prediction, a multiscale CNN model is proposed which uses multiscale feature images to obtain multiscale CNN features in the classification of LiDAR point clouds. It is known that human vision is a multiscale process [36]. By changing the width of cell *w*, different scales of feature images can be generated for the same point. Different scales of feature images can help us get both robust semantic information and precise location information, thus enriching the features for classifying point clouds.

**Figure 4:**
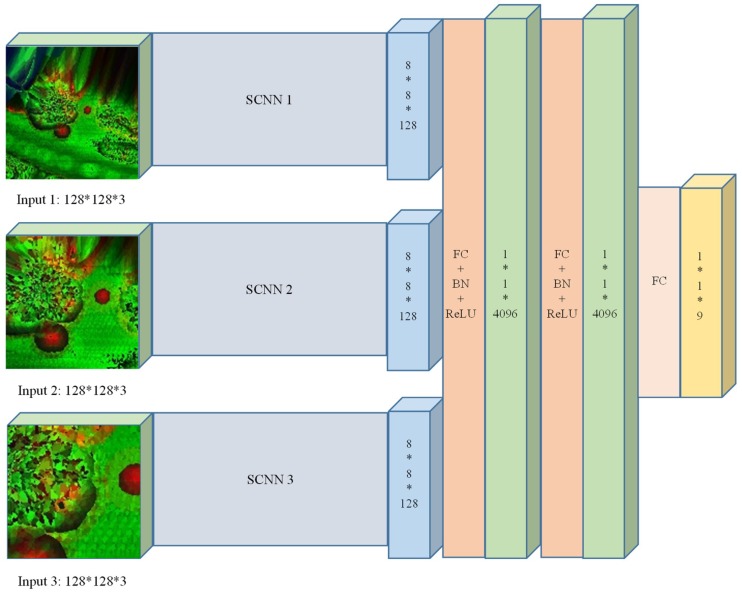
The architecture of the MCNN.

Our architecture of multiscale CNN is shown in Figure 4. Different scales of features were extracted by independent CNNs and fused into multiscale features to make the prediction. Since we get the different scale features by change the width of the cell, all the feature images have the same size (128 × 128 × 3). CNN models of different input scales were named SCNN1, SCNN2, and SCNN3. Both have the same architecture, as shown in Figure 3. In our work, feature images were classified into nine categories: power-line, low vegetation, impervious surfaces, car, fence/hedge, roof, facade, shrub, and tree. Thus, our multiscale CNN has nine outputs.

FC denotes the fully connected layer. All the neurons in the previous layer were connected with every single neuron in the latter layer. In the last fully connected layer, a standard feed-forward manner was operated to obtain the label prediction result [37]. The number of the classes to be identified was *N*. A vector, *ρ*, is computed, and its elements, *ρ_k_*, encode the probability mass function over the N classes as:

(6) $$\rho_{k}=p(y=k|x)=\frac{\exp(a_{k}(x))}{\sum_{j}^{N}\exp(a_{j}(x))}$$

x denotes the input of the images, $a_{j}(x)$ is the *j*^th^ unit at the output layer. and *k* denotes the class index. From the probabilities, the most probable class was estimated as:

(7) $$\hat{y}=\underset{k}{\mathrm{argmax}}p(y=k|x)$$

All parameters in the MCNN were determined automatically from the training images. If the training dataset has *N_s_* sample images with corresponding ground-truth labels, the error function can be defined as:

(8) $$\frac{1}{N_{s}}\sum_{i=1}^{N_{s}}f(\rho^{i},y_{true}^{i})+\lambda {\left\|w\right\|}_{2}$$

In this paper, f(.,.) was defined as the cross-entropy error function [38] that evaluates the agreement between the network’s output, $\rho^{i}$, and the ground-truth label, $y_{true}^{i}$.w is the vector including all weights of the network, ${\left\|•\right\|}_{2}$ denotes the L2 norm, and λ regulates the influence of the magnitude of the weight vector on the error function. The approximate solution was obtained using the stochastic gradient descent (SGD) [11] method during backpropagation.

In order to fully train the MCNN, the parameters of the MCNN were initialized with the parameters of SCNN separately, and the fine-tuning of the MCNN was used to complete the training.

### 3.4. Workflow

**Figure 5:**
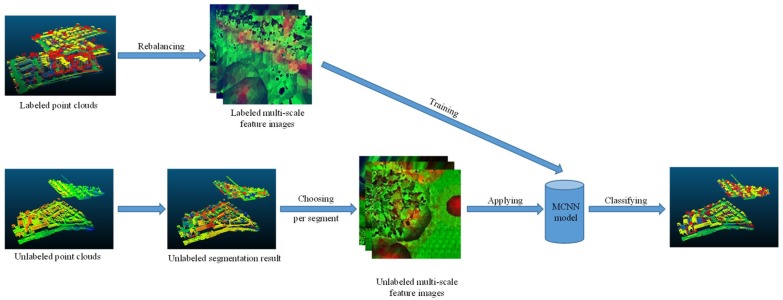
The workflow of the proposed method.

The workflow of the proposed method is shown in Figure 5. In order to reduce the data redundancy and balance the number of points in each category, a class rebalancing strategy was used for the training data. If the point number of one category was larger than a certain size, we reduced it. If not, we kept it unmodified. During the training period, the MCNN model was trained using the multi-scale feature images generated from the rebalanced training data. As for the testing data, we initially applied the three-step region-growing method to obtain the segmentation results. Then, a voting strategy was used. We randomly chose several points in each segment to generate the multi-scale feature images. In the testing period, the most frequent label in the points predicted by the MCNN was assigned to the segment.

## 4. Experimental Results

### 4.1. Test Data

To evaluate our approach, we set the experiments using the ISPRS 3D labelling benchmark. This dataset has been presented in the scope of the ISPRS Test Project on Urban Classification and 3D Building Reconstruction and simultaneously serves as the benchmark dataset for the ISPRS benchmarks on 2D and 3D semantic labeling. The ALS data was acquired using a Leica ALS50 system with 45° field of view and a mean flying height 500 m above ground over Vaihigen, a small village in Germany [39]. The point density of the data was between 10 and 20 points/m^2^. For the semantic labeling task, nine classes (i.e., power, low vegetation, impervious surface, car, fence/hedge, roof, facade, shrub, and tree) were labeled by the authors of [17]. Point in the ALS data contained the spatial XYZ-coordinates, intensity values, and the number of returns. The given data was subdivided into two areas. The first area was the training area containing 753,876 labeled points. The nine categories were power-line, low vegetation, impervious surfaces, car, fence/hedge, roof, facade, shrub, and tree. The second area was the testing area containing 411,722 unlabeled points. More detailed information is shown in Table 2. For the trainging dataset, a class rebalancing strategy was applied. We used the same class rebalancing strategy as the one used in [6]. If the point number was larger than 15,000, we rebalanced it. If not, it remained unmodified.

The quantity of the classification result was evaluated based on the ISPRS contest criteria: precision/correctness, recall/completeness, F1 score, and the overall accuracy. The tp (true positive), fp (false positive), and the fn (false negative) values for each category were calculated. The precision value, recall value, and F1 score were calculated as follows:

(9) $$\{\begin{array}{c} precision=\frac{tp}{tp+fp}\\ recall=\frac{tp}{tp+fn} \end{array}$$

(10) $$F_{1}=2\times \frac{precision\times recall}{precision+recall}$$

### 4.2. Experiment Results

The experiment focused on the influence of segmentation and CNN architecture in classification results and testing efficiency. Five strategies were used in the experiment:

- Point_S indicates the method used in [6]. It is a point-based method and uses the SCNN for semantic labeling.
- Point_M replaces the SCNN in Point_C with the MCNN.
- SegS_M adds the simple normal vector-based region-growing segmentation strategy into Point_M.
- SegT_M adds our three-step region growing segmentation strategy into Point_M.
- SegT_S replaces the MCNN in SegT_M with the SCNN.

**Figure 6:**
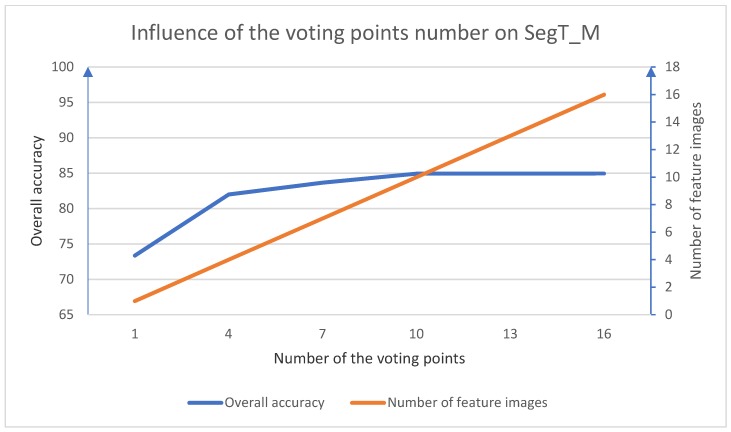
The influence of the voting point number on SegT_M.

As for testing procedure, a voting strategy was used. We randomly chose several points in each segment to generate feature images. For each point, three multi-scale feature images were generated. Based on the discussion in [6], we set the cell width of the feature images as 0.05, 0.1, and 0.2 m. For the region growing steps, we set the normal vector threshold to 5°, the intensity threshold to 10, and the planarity threshold to 0.15. The training of the CNN model was performed on a Dell T630 PC with an Intel Xeon E5-2603v3 CPU, 64GB RAM, and an NVIDIA Tesla K20c. The professional graphic card helped us to save time in calculating the MCNN parameters. The influence of the voting point number on SegT_M is shown in Figure 6. Taking the computational efficiency and the overall accuracy into consideration, we chose 10 points in each segment generating the feature images. The per-class accuracy and the overall accuracy for each method are shown in Table 3. The running time for each step, the number of feature images, the overall accuracy (OA), and the average F1 scores of each method are shown in Table 4.

### 4.3. ISPRS Benchmark Testing Results

To evaluate our method performance against others, the results of the classification were submitted to the ISPRS organizers for evaluation. The per-class accuracy and the overall accuracy for each submission are shown in Table 5, and the per-class F1 score and the average F1 score for each submission are shown in Table 6. In ISS_7 [40], supervoxel-based segmentation and the color-based region-growing segmentation were used to segment the ALS data. A machine learning algorithm was used to label these segments. In UM [41], an OvO (one-vs-one) machine learning strategy was applied to obtain a 3D semantic labeling result. Features extracted from LiDAR point-attributes, textural analysis, and geometric attributes were used. In HM_1 [42], a conditional random field method which used a random forest classifier for generating the unary potentials and a variety of the contrast-sensitive Potts models for generating the pairwise potentials was used for the point-based semantical labelling task. In WhuY3 [6], a point-based semantic labeling method using a convolutional neural network was proposed. This point-based feature image generation method transformed the 3D neighborhood features of a point into a 2D image. In LUH [26], a two-layer hierarchical framework was used for the contextual classification of LiDAR data. The supervised approach classified points and segments prospectively with two independent conditional random fields. In RIT_1 [23], 3D-coordinated and three corresponding spectral features for each point were used by a 1D-fully convolutional neural network to generate point-wise labeling while learning contextual features in an end-to-end fashion.

## 5. Discussion

The original purpose of our proposed method was to reduce the computation burden of the point-based method in our previous work [6]. As shown in Table 3 and Table 4, the three-step region-growing segmentation strategy has a good performance compared with the MCNN. Although the MCNN needs more feature images, it did improve the overall accuracy and the average F1 score of the classification result (e.g., comparing Point_S with Point_M and SegT_S with SegT_M). The time efficiency of the framework was closely related to the number of test feature images. The segmentation-based strategies reduced the test feature images completely. The total test feature image number of SegT_M was only one tenth that of Point_M and one fifth that of SegS_M. Although the segmentation method may cause some time, the testing efficiency of the framework is indeed improved. The overall accuracy and the average F1 score also have a good result.

**Figure 7:**
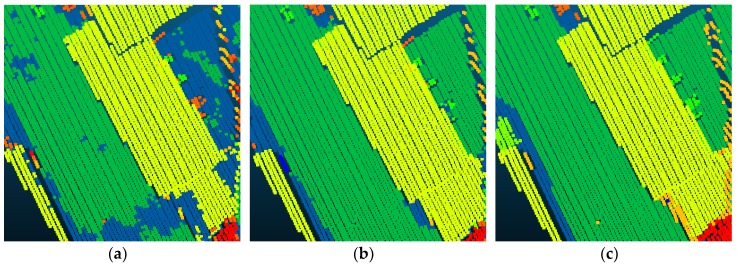
The detailed improvement of low vegetation and impervious surfaces classification. (**a**) The Point_S result. (**b**) The SegT_M result. (**c**) The ground truth.

**Figure 8:**
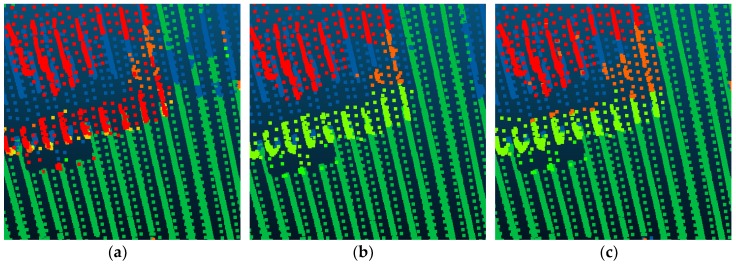
The detailed improvement of fence/hedge classification. (**a**) The Point_S result. (**b**) The SegT_M result. (**c**) The ground truth.

**Figure 9:**
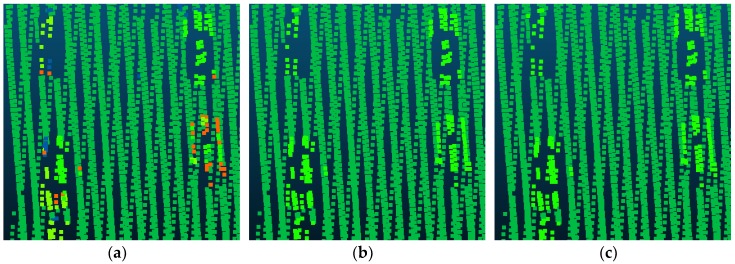
The detailed improvement of car classification. (**a**) The Point_S result. (**b**) The SegT_M result. (**c**) The ground truth.

**Figure 10:**
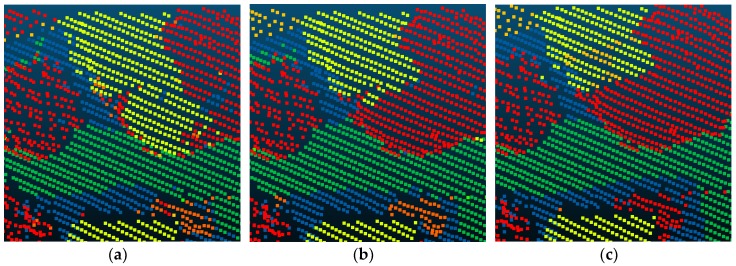
The detailed improvement of roof and tree classification. (**a**) The Point_S result. (**b**) The SegT_M result. (**c**) The ground truth.

Compared with Point_S [6], our method shows a better classification performance. For the large planar objects, such as low vegetation, impervious surfaces, and roofs, the mistaken mix of different categories was solved. As shown in Figure 7, since the planarity of the low vegetation and the impervious surfaces had little differences, these classes may be mixed together in a point-based classification method. Basing on the intensity values and the normal angle differences, these objects have been clustered together. Compared with Point_S, our method (SegT_M) improved the results of categorizing low vegetation (+3.3%) and impervious surfaces (+0.5%). For the small non-planar objects, such as fences, hedges and cars, our method has better classification results over the point-based method. As shown in Figure 8 and Figure 9, the point-based method may misclassify fences, hedges, and cars into shrubs and other classes. In the second and third steps of our segmentation, planarity values were used to cluster these objects. The whole segment served as an integer for the following classification procedure. Compared with Point_S, our method (SegT_M) improved the results of classifying the fence/hedge (+27.8%) and car (+9.6%) categories. In some areas, as shown in Figure 10, trees and roofs are hard to distinguish because of the unusual distribution of their points and the similarity of their planarity. The segmentation-based strategy can solve this problem to some degree. Compared with the Point_S, our method (SegT_M) improved the classification of the tree category (+5.2%).

**Figure 11:**
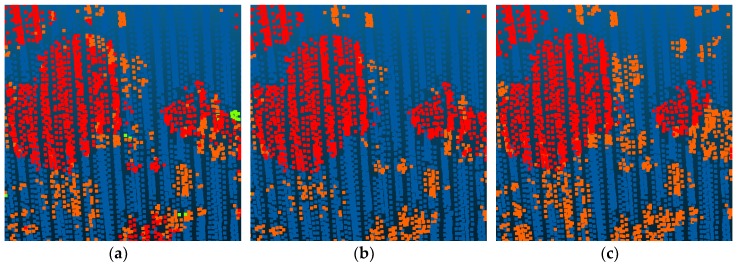
The failed shrub and tree classification. (**a**) The Point_S result. (**b**) The SegT_M result. (**c**) The ground truth.

As shown in Table 5 and Table 6, our method had a satisfactory performance over all participants on the ISPRS WG II/4 Vaihingen 3D Semantic Labeling task. Its overall accuracy and the average F1 score were ranked 1st of all participants. Based on our three-step segmentation strategy, planar objects, such as low vegetation (ranking 1st), impervious surfaces (2nd), and roofs (1st), and smaller objects, such as cars (1st), fences/hedges (1st), and shrubs (1st), had a good performance in F1 score. The multi-scale convolutional neural networks exploited the potential of the selected features, as we expected. There were also some misclassifications in our final result. As shown in Figure 11, shrubs and low vegetation were difficult to distinguish. Some shrubs were mixed up with trees and low vegetation. This is because, in the segmentation procedure, these points were clustered into the same segment. Our method needed to adjust several parameters in the segmentation step. It was hard to make the segmentation result suit all the categories. A more automatic and universal segmentation method should be proposed. Furthermore, only LiDAR was used in our experiments. In order to improve our further classification performance, the corresponding orthoimages could be used in our future work.

## 6. Conclusions

In this paper, we propose a three-step region growing segmentation method for segment-based point cloud classification. The three-step strategy minimized both under-segmentation and over-segmentation and provides a good starting point for the following procedure. The computational burden was reduced. Then, a multi-scale convolutional neural model was used to train and classify the feature images. Based on the voting strategy, each segment can be classified into nine classes by the multi-scale convolutional neural model. The classification result had a satisfactory performance on the ISPRS dataset compared with state-of-the-art methods. As shown in Table 5 and Table 6, the overall accuracy and the average F1 score rank the first compared with the other considered approached.

Our method still has the potential for improved performance. One such room for improvement is that the results of the segmentation can further influence the classification output. The complex parameter setting makes it hard to obtain the best result. In future works, we will propose a more automatic and universal segmentation method to solve this problem. Another room for improvement is that only LiDAR is used in our current method. To further improve our classification performance and apply our method to more complex 3D classification tasks, corresponding image data will be used in our future work.

## Figures and Tables

**Table 1:** LiDAR features for classification.

Type	Symbol	Feature
Height features	Δz	Height above DTM
Echo features	I	Intensity
Eigenvalue features	$P_{\lambda }$	Planarity
	$S_{\lambda }$	Sphericity
Local plane features	$\delta_{n_{v}}^{2}$	Variance of deviation angles

**Table 2:** Number of 3D points per class.

Class	Training Set	Rebalancing Result	Test Set
Powerline	546	546	N/A
Low Vegetation	180,850	18,005	N/A
Impervious Surfaces	193,723	19,516	N/A
Car	4614	4614	N/A
Fence/Hedge	12,070	12,070	N/A
Roof	152,045	15,235	N/A
Facade	27,250	13,731	N/A
Shrub	47,605	11,850	N/A
Tree	135,173	13,492	N/A
∑	753,876	109,059	411,722

**Table 3:** Per-class accuracy and the overall accuracy of each strategy.

Method	Power	Low Vegetation	Impervious Surface	Car	Fence/Hedge	Roof	Facade	Shrub	Tree	OA
**Point_S**	24.7	81.8	91.9	69.3	14.7	95.4	40.9	38.2	78.5	82.3
**Point_M**	25.2	83.1	92.1	71.2	19.3	95.5	42.1	39.2	79.3	83.0
**SegS_M**	28.3	84.7	92.5	69.5	18.7	95.5	40.7	38.3	78.4	83.3
**SegT_S**	26.8	84.3	91.2	71.2	33.7	95.4	43.3	43.6	81.2	83.6
**SegT_M**	31.2	85.0	92.4	78.9	42.5	95.6	46.5	42.4	83.7	84.9

**Table 4:** Comparison of the computation time for each strategy.

	Point_S	Point_M	SegS_M	SegT_S	SegT_M
**Segmentation time (min)**	0	0	4:20	7:40	7:40
**Number of training feature images**	109,059	327,177	327,177	109,059	327,177
**Training feature images generation time (h)**	0.4	1.3	1.3	0.4	1.3
**Number of testing feature images**	411,722	1,235,166	538,398	39,430	118,290
**Testing feature images generation time (h)**	1.6	4.7	2.0	0.2	0.5
**Training time (h)**	6.5	20.0	20.0	6.5	20.1
**Testing time (s)**	70.4	172.8	83.8	10.4	30.7
**Overall Accuracy (%)**	82.3	83.0	83.3	83.6	84.9
**Average F1 (%)**	61.6	63.7	65.7	64.3	69.2

**Table 5:** A quantitative comparison between the per-class accuracy and the overall accuracy of our method and other published methods on the ISPRS test set.

Method	Power	Low Vegetation	Impervious Surface	Car	Fence/Hedge	Roof	Facade	Shrub	Tree	OA
**ISS_7**	40.8	49.9	96.5	46.7	39.5	96.2	-	52.0	68.8	76.2
**UM**	33.3	79.5	90.3	32.5	2.9	90.5	43.7	43.3	85.2	80.8
**HM_1**	82.8	65.9	94.2	67.1	25.2	91.5	49.0	62.7	82.6	80.5
**WhuY3**	24.7	81.8	91.9	69.3	14.7	95.4	40.9	38.2	78.5	82.3
**LUH**	53.2	72.7	90.4	63.3	25.9	91.3	60.7	73.4	79.1	81.6
**RIT_1**	29.8	69.8	93.6	77.0	10.4	92.9	47.4	73.4	79.3	81.6
**Ours**	31.2	85.0	92.4	78.9	42.5	95.6	46.5	42.4	83.7	84.9

**Table 6:** A quantitative comparison between the per-class F1 score and the average value of our method and other published methods on the ISPRS test set.

Method	Power	Low Vegetation	Impervious Surface	Car	Fence/Hedge	Roof	Facade	Shrub	Tree	Avg. F1
**ISS_7**	54.4	65.2	85.0	57.9	28.9	90.9	-	39.5	75.6	55.27
**UM**	46.1	79.0	89.1	47.7	5.2	92.0	52.7	40.9	77.9	58.96
**HM_1**	69.8	73.8	91.5	58.2	29.9	91.6	54.7	47.8	80.2	66.39
**WhuY3**	37.1	81.4	90.1	63.4	23.9	93.4	47.5	39.9	78.0	61.63
**LUH**	59.6	77.5	91.1	73.1	34.0	94.2	56.3	46.6	83.1	68.39
**RIT_1**	37.5	77.9	91.5	73.4	18.0	94.0	49.3	45.9	82.5	63.33
**Ours**	42.5	82.7	91.4	74.7	53.7	94.3	53.1	47.9	82.8	69.2
